# Monitoring Citrus Soil Moisture and Nutrients Using an IoT Based System

**DOI:** 10.3390/s17030447

**Published:** 2017-02-23

**Authors:** Xueyan Zhang, Jianwu Zhang, Lin Li, Yuzhu Zhang, Guocai Yang

**Affiliations:** 1 School of Computer and Information Science, Southwest University, Chongqing 400715, China; zxyssyssy@email.swu.edu.cn (X.Z.); cqkxxn@163.com (L.L.); yuzhuzane@163.com (Y.Z.); 2 Dean’s Office, Tianjin Railway Technical and Vocational College, Tianjin 300240, China; zjw63@163.com

**Keywords:** Internet of Things technology, single-point multi-layer detection, fertilization and irrigation decision support

## Abstract

Chongqing mountain citrus orchard is one of the main origins of Chinese citrus. Its planting terrain is complex and soil parent material is diverse. Currently, the citrus fertilization, irrigation and other management processes still have great blindness. They usually use the same pattern and the same formula rather than considering the orchard terrain features, soil differences, species characteristics and the state of tree growth. With the help of the ZigBee technology, artificial intelligence and decision support technology, this paper has developed the research on the application technology of agricultural Internet of Things for real-time monitoring of citrus soil moisture and nutrients as well as the research on the integration of fertilization and irrigation decision support system. Some achievements were obtained including single-point multi-layer citrus soil temperature and humidity detection wireless sensor nodes and citrus precision fertilization and irrigation management decision support system. They were applied in citrus base in the Three Gorges Reservoir Area. The results showed that the system could help the grower to scientifically fertilize or irrigate, improve the precision operation level of citrus production, reduce the labor cost and reduce the pollution caused by chemical fertilizer.

## 1. Introduction

Informatization is the sign and key of agricultural modernization. Agricultural information can significantly change small scale of agricultural production, great temporal and spatial variation, low scale merit and other industrial weakness. Moreover, it plays an important role in the development of agriculture and the full realization of a well-off society [1,2]. The Internet of Things (IoT) is defined as things connected to things in the Internet [3,4,5]. The agricultural Internet of Things is a new trend in world agricultural development, a new type of agriculture which combines the Internet of Things and agricultural production [6,7,8]. It will bring agriculture into the digital information age [9]. Agricultural Internet of Things is able to implement digital design, intelligent control, precise operation and scientific management for various agricultural elements. So it achieves a comprehensive perception, reliable transmission and intelligent processing, and ultimately achieves high yield, high efficiency, high quality, ecological and safety purposes [10].

Citrus is largest fruit crop in the world. Its planting area and output are the first of the fruit trees. In addition, it is the world’s third largest trade agricultural products. Citrus is produced in 135 countries and regions all over the world. Among these areas, China’s citrus planting area and production is in the first place in the world, becoming the world’s largest citrus producing country. In China, most citrus management methods are still in the relatively backward stage, monitoring of water, nutrients, and the temperature is still in the manual monitoring phase [11]. They result in inconvenient operation, time-consuming and laborious, low precision and the lack of information. Furthermore, the data acquisition will have a certain delay. So managers cannot achieve comprehensive and clear grasp of the orchard information [12]. Based on this, the paper applies the Internet of Things technology and single-point multi-layer detection method to the soil moisture, temperature and nutrient monitoring, sets up citrus orchard fertilization irrigation expert knowledge base and makes expert decision according to the soil condition in real time to guide the actual production process of citrus. The emergence of wireless sensor, data fusion and internet technology, can achieve the remote automatic monitoring on the citrus water, nutrient and temperature of growth environment. And through the model analysis and data processing, expert decision-making system can give effective measures of citrus management. Practice has proved that single-point multi-layer detection technology can effectively expand the detection range of citrus growth environment, and it is helpful to construct a more accurate expert knowledge base. At the same time, the system can effectively guide fruit growers to scientific management of citrus orchards and significantly improve the yield of citrus.

## 2. Overall Structure

### 2.1. System Design Objectives

The real-time monitoring system of citrus soil moisture and nutrient is designed to monitor the soil moisture and nutrient status in the citrus orchard, so that fruit farmers can grasp the condition of orchard in time, and under the guidance of decision-making support system adjust fertilization irrigation strategy. This determines the design objectives of the system.

1. Put forward the scientific distribution technology of the soil nutrient and moisture real-time monitoring system in mountain citrus orchard. It makes the layout more reasonable and the monitoring area is effectively covered.
2. Research and develop scientific detection methods to obtain more accurate citrus growth environment conditions.
3. Integrate wireless sensor network of citrus soil nutrient and moisture and remote information management system and intelligent decision support system based on ZigBee technology [13,14]. Finally, carry on scientific experimental demonstration.

### 2.2. System Structure

**Figure 1:**
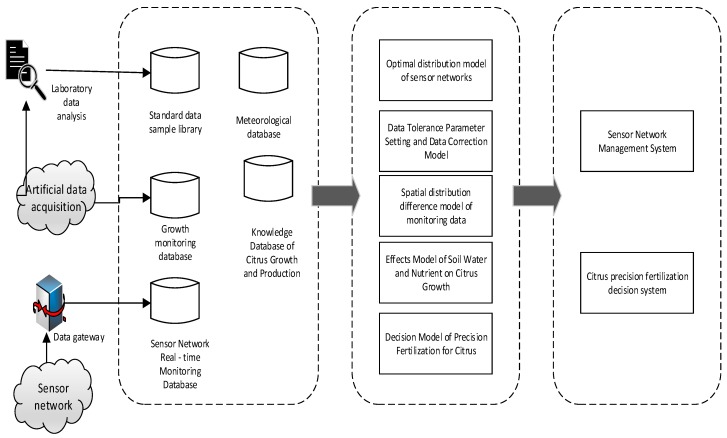
Overall system architecture diagram.

The IoT platform design idea is applied to the real-time monitoring system of citrus soil moisture and nutrient. The system is divided into four layers: perception layer, network transmission layer, information service layer and application layer. The overall system structure is shown in Figure 1.

Perception layer: Perception layer is mainly to achieve data acquisition and perception [15,16] including citrus soil moisture and temperature, air humidity and temperature, soil nutrients and sensor space coordinate. According to the experiment, we found that the temperature and humidity at different soil depths at the same location can better reflect the environmental condition. So we use single-point multi-layer detection method and the soil depth of 20 cm, 40 cm and 60 cm was selected for monitoring. Soil moisture is measured by a special soil moisture sensor which can realize on-line detection. The output voltage of the sensor varies with the change of soil water content. Air temperature and humidity are measured using SHT17 digital temperature and humidity sensors (Sensirion, Zurich, Switzerland). There is no suitable on-line soil nutrient detection sensor. Nutrient change is slow and the measurement takes a long period of time. Therefore, the portable soil nutrient detector is selected to detect soil nutrients.

Network transmission layer: It mainly includes the citrus orchard site wireless sensor network and data transmission facilities connected to the Internet. The data transmission network includes the short-distance transmission part of the citrus orchard and the data long-distance transmission part. Internet of Things Gateway is the core of wireless sensor network equipment which can achieve connection with the public network and protocol conversion. The short distance transmission in the citrus orchard uses ZigBee wireless communication technology and ad hoc network technology [17,18], which has the advantages of low power consumption, strong mobility and other advantages in deployment and maintenance. The long-distance transmission uses GPRS [19,20] to connect to the Internet server, which supports IPV4 and IPV6 on the transmission protocol. The concepts of rumor and gossip routing algorithms are widely employed in sensor networks [21] and ad hoc networks [22]. So we also use this method in our system. The main function of this layer is to transmit various agricultural information collected by the perception layer to the background network server through the network.

Information service layer: This layer mainly includes hardware and software. The hardware part adopts PC cluster control and the local area network. The databases include the standard data sample database, the sensor network monitoring database, the citrus production database, the meteorological data and the citrus production domain knowledge database. These data provide information support services for the application layer.

Application layer: it is an integrated information platform based on business logic. The software running on the platform include a sensor network management system, a WEB GIS-based monitoring data query and analysis system, and a citrus precision fertilization decision support system. Sensor network management system proposes optimal layout recommendations based on the citrus orchard sensor network optimization distribution model. The data query and analysis system based on WEB GIS can get the humidity, temperature and nutrient value of citrus soil. The data are obtained from the real-time monitoring database of the sensor network in the GIS spatial database through data fault correction. According to soil moisture and nutrient status, fruit growers use citrus growth model and citrus precision fertilization and irrigation support model to obtain the citrus fertilization irrigation support decision.

## 3. Design Scheme

### 3.1. System Hardware Design

#### 3.1.1. Sensor Control Node

**Figure 2:**
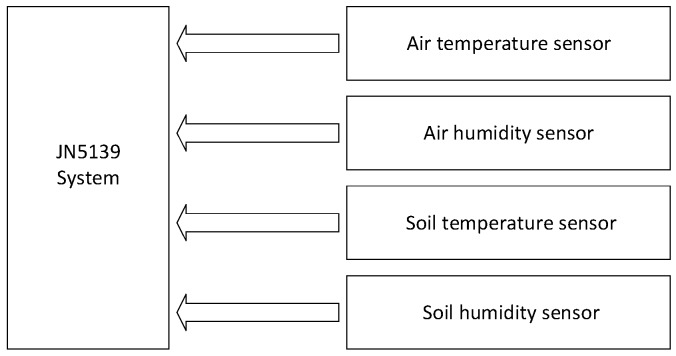
Sensor nodes structure.

The main task of the sensor control node is to collect the citrus orchard soil temperature and humidity, air temperature and humidity and other parameters, and transfer data to the master node. We select HA2002 (Handan Dingrui Electronics Co., Ltd, Handan, China) as the soil temperature sensor, HA2001 (Handan Dingrui Electronics Co., Ltd, Handan, China) as the soil moisture sensor, and the type of air temperature and humidity sensor is FM-KWS (Hebei fly dream Electronic Technology Co., Ltd, Handan, China). These sensors have the same characteristics: fast response time, high accuracy, wide range, good stability. JN5139 module is a series of surface mount modules that enable users to realize IEEE802.15.4 or ZigBee compatible system in the shortest time and at the lowest cost. The field wireless sensor can send the parameters to the JN5139 control node through the ZigBee. The sensor node structure is shown in Figure 2.

**Figure 3:**
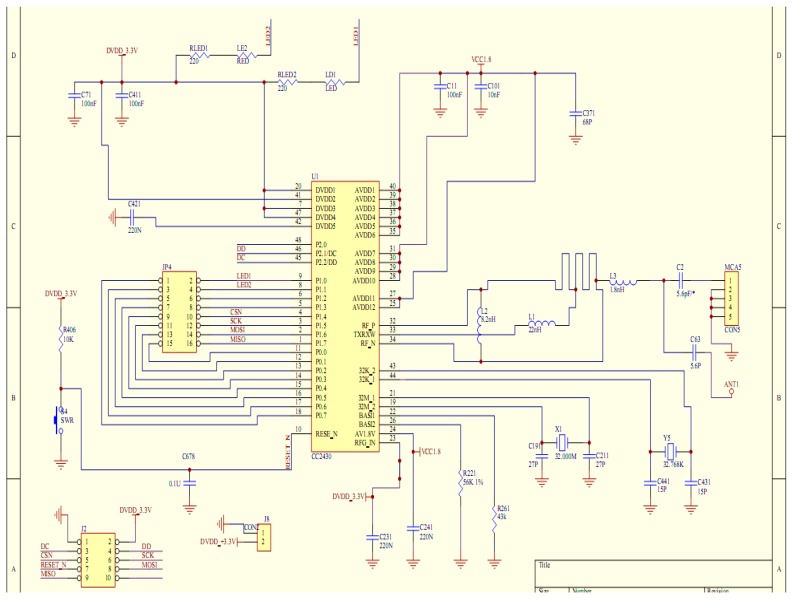
Part of the circuit diagram of the sensor node.

Terminal node part of the circuit diagram is shown in Figure 3.

#### 3.1.2. Field Master Control Node

**Figure 4:**
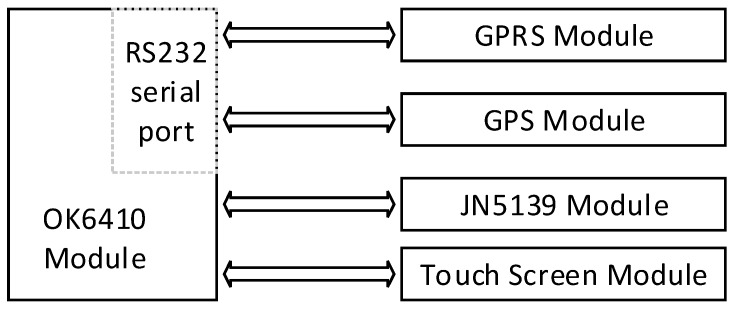
Spot main control node structure.

The system uses ARM11-based S3C6410 (YoujianhengtianTechnology Co., Ltd, Shenzhen, China) [23] as the master chip, using FORLINX’s OK6410 (Forlinx Embedded Tech. Co., Ltd, Baoding, China) control board module. The GPRS module adopts HUAWEI GTM900C (Huawei Technologies Co., Ltd., Shenzhen, China) and the GPS module uses Trimble 4600LS single frequency GPS receiver. The receiver is produced by the world’s largest GPS manufacturer Trimble Navigation Company (Sunnyvale, CA, USA), and it is the first integrated GPS receiver which has high quality, high precision, high efficiency and low price. The ARM module sent the AT commands to the GTM900C module through the serial port. Through this method, the GTM900C module achieves the corresponding function. 4600LS receives the geographical coordinates of space and transmits them to the control board through the RS232 serial port. OK6410 connects with the JN5139 GPRS module GTM900 and 4600LS through the USB serial port. The field controller node is the sink node of the sensor network which monitors the site. It is responsible for managing and maintaining the ZigBee network. The field control unit has a corresponding LCD touch screen, real-time display dynamic changes of soil temperature and humidity, air temperature and humidity in collection points. The structure of the field control node is shown in Figure 4.

### 3.2. System Software Design

**Figure 5:**
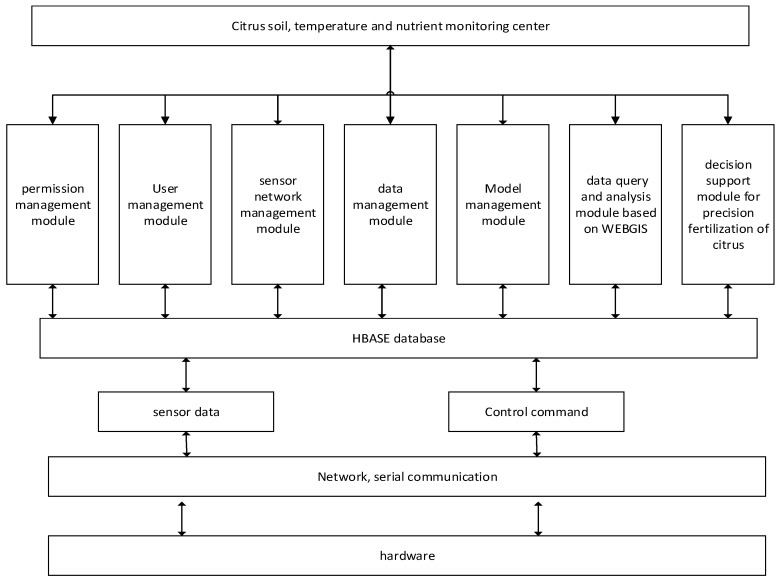
Software architecture of citrus soil temperature and humidity monitoring system.

Citrus soil, temperature and nutrient monitoring system is the core of the system. Its architecture is shown in Figure 5 below.

In this section, the permission management module can assign different permissions to different roles. In the sensor network management module, users can manage sensor network nodes and view the sensor status. The data query and analysis module based on WEB GIS can check the spatial distribution of soil moisture, temperature and nutrient. The decision-making system in the decision support module of citrus precision fertilization can make decisions according to the soil moisture and temperature. If the system decision-making model is not ideal, it will be modified or replaced to adjust the expert decision-making advice in a short time. It is the management module function of the decision model. Data management mainly includes data receiving, data storage and data processing. In order to make users more intuitive to observe the effects of temperature, humidity and nutrient and other parameters on citrus growth, providing the basis for scientific cultivation, citrus soil temperature and nutrient monitoring center drawn curves for different functions on the sensor upload data. Users can query the environmental parameters monitored by the sensor nodes at different locations and at different times.

#### Decision Support Module

**Figure 6:**
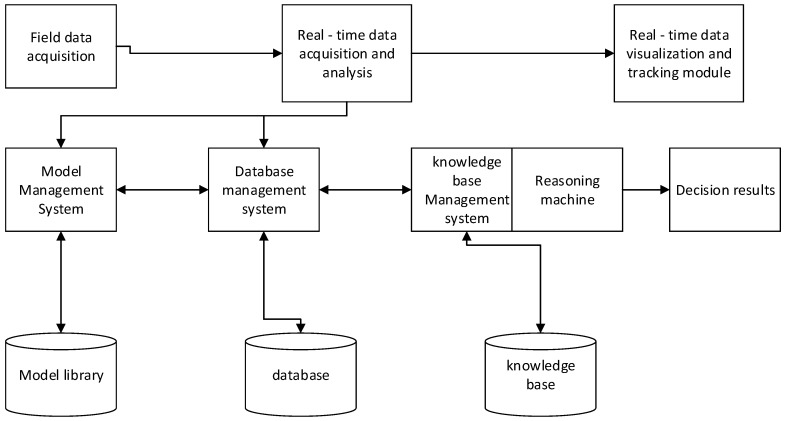
Decision system procedures.

The decision support module is one of the most important parts of the system, and other modules provide supports for it. The model management module includes the expert knowledge base of citrus fertilization and irrigation, and allows the authorized users to revise and supplement the knowledge base according to the actual situation. The data management module processes and analyzes the collected data. It ensures that these data meet the data input format of the model. We use the single-point multi-layer detection method when using sensors to obtain soil moisture, temperature and other environmental conditions. The application of this method makes the grower have a more comprehensive and accurate grasp of citrus growth environment status, while improving the accuracy of fertilizer and irrigation decisions. Take the citrus orchard soil moisture monitoring as an example, for the same fruit tree the water content at 20 cm, 40 cm and 60 cm below the surface will be monitored. The three humidity values of different surface depths are averaged as one of the input conditions of the irrigation decision system. Table 1 shows some examples of expert knowledge bases for citrus irrigation in sandy soil types. An irrigation decision-making system based on soil properties, the average water content and time stamp gives reasonable irrigation decisions. The citrus intelligent irrigation decision-making system process is shown in Figure 6.

## 4. System Testing

**Figure 7:**
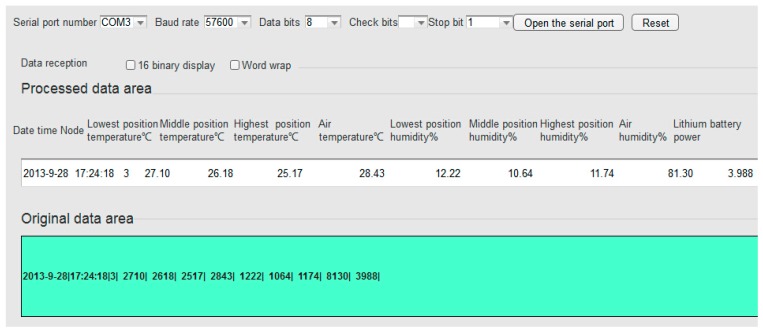
Remote server monitoring interface.

The server receives soil temperature and humidity, air temperature and humidity every minute. Soil temperature and humidity, air temperature and humidity sensors transmit data to the network through the RS232 serial port. The interface of remote server receiving data is shown in Figure 7.

**Figure 8:**
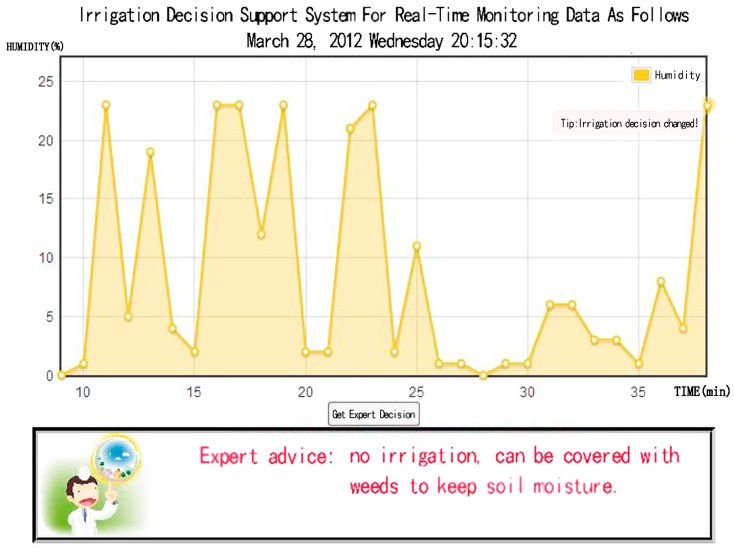
Irrigation decision support interface.

The data collected by citrus soil temperature, humidity and air temperature, humidity sensor will be transmitted to the background. They need to be compared with the standard sample database and modified using data tolerance and data correction model to get the desired data format. The citrus precision fertilization and irrigation support model and citrus soil moisture, temperature and nutrient monitoring model give real-time expert decision-making advice according to the citrus growth knowledge database. The soil nutrients will not change significantly in a certain period of time and there is no suitable wireless sensor. So the system uses artificial data collection. The real time monitoring part mainly monitors the temperature and humidity of soil and air and gives irrigation decisions in real time based on air temperature and humidity. Figure 8 is the interface of the irrigation decision support system. The corresponding season is spring, when the average soil moisture is more than 20%. The system suggests that the orchard does not need to be irrigated, and the experts reached the same conclusion as the system after their field visit. After a year of use we found that the soluble solid content of fruit was increased by 1%–2%, orchard per mu yield increased 500 kg or more and save water and fertilizer resources 20%. It proves that the system can guide the management of the citrus orchard in the Three Gorges reservoir area scientifically. 

## 5. Conclusions 

In this study, the project of the citrus moisture, temperature and nutrient monitoring based on the Internet of Things platform was proposed. The project regards early warning and decision-making as the basic objectives, and provide a reference solution for citrus large-scale cultivation. As a result of the hierarchical thinking of the Internet of Things, the decision support system is divided into the perception layer, network transport layer, information service layer, application layer. This idea reduces the coupling between various services and improves the reliability of the system. The single-point multi-layer detection method to obtain temperature, humidity and nutrients is an innovative point of the system. This will not only expand the detection range, but also improve the accuracy of the model. According to the characteristics of geographical environment and citrus management experience of many years, the expert knowledge base suitable for the Three Gorges reservoir area was established, which provided a model for citrus fertilization and irrigation decision support. Practice has proved that the system can make scientific management decisions according to citrus growth conditions. The system has many shortcomings, such as “silo” solutions [24] and high cost problems. In the future we will optimize it in these areas.

## Figures and Tables

**Table 1:** Knowledge base of citrus irrigation (sandy soil).

Soil Properties	Humidity (%)	Season	Diagnostic Conclusion	Expert Advice
sandy soil	<17	spring	lack of water	Immediately carried out winter irrigation. first build the hillock in the crown to form a water plate, Irrigate 20 kg per square meter of canopy projected area. Dug irrigation water hole 2–4 in the drip line around the crown; Irrigate 10 kg per square meter of canopy projected area.
	<15	autumn winter		Immediately carried out winter irrigation. first build the hillock in the crown to form a water plate, Irrigate 10 kg per square meter of canopy projected area. Dug irrigation water hole 2–4 in the drip line around the crown;Irrigate 7 kg per square meter of canopy projected area. enough water is given to the tree tray before the frost.
	<18	summer		First loose soil and cover tree plate with grass. Irrigate 30 kg per square meter of canopy projected area. Dug irrigation water hole 2–4 in the drip line around the crown; Irrigate 30 kg per square meter of canopy projected area.
	16–20	spring		First loose soil and cover tree plate with grass. Irrigate 15 kg per square meter of canopy projected area. Dug irrigation water hole 2–4 in the drip line around the crown. Irrigate 10 kg per square meter of canopy projected area.
	15–20	autumn winter	low level	Immediately carried out winter irrigation. first build the hillock in the crown to form a water plate, Dug irrigation water hole 2–4 in the drip line around the crown. Irrigate 15 kg per square meter of canopy projected area. enough water is given to the tree tray before the frost.
	17–21	summer		First loose soil and cover tree plate with grass. Irrigate 20 kg per square meter of canopy projected area. Dug irrigation water hole 2–4 in the drip line around the crown. Irrigate 15 kg per square meter of canopy projected area.
	21–80	anniversary	suitable	Without irrigation, soil cover and weed moisture under the canopy.
	>80	anniversary	excess	Timely excavate ditches and discharge orchard water. If there is an unbroken spell of wet weather, plastic film can be used for ground cover.
